# Neutrophils Promote Larynx Squamous Cell Carcinoma Progression via Activating the IL-17/JAK/STAT3 Pathway

**DOI:** 10.1155/2021/8078646

**Published:** 2021-12-13

**Authors:** Tianyi Liu, Shimin Zong, Yang Jiang, Rui Zhao, Jie Wang, Qingquan Hua

**Affiliations:** ^1^Department of Otorhinolaryngology, Renmin Hospital of Wuhan University, Wuhan, 430060 Hubei, China; ^2^Department of Otorhinolaryngology, Union Hospital, Tongji Medical College, Huazhong University of Science and Technology, Wuhan 430022, China

## Abstract

Laryngeal squamous cell carcinoma (LSCC) is the main type of laryngeal cancer with poor prognosis. Incidence of LSCC increases every year, posing a great threat to human health. The underlying mechanism needs further study. Neutrophils are the most prevalent type of immune cells, which play vital roles in crosstalk between the microenvironment and cancer cells. In our study, we aim to figure out the complex regulation between neutrophils and LSCC. Our experiments showed that LSCC cells could promote the activation and mobility of neutrophils. And, in return, neutrophils enhanced the proliferation, migration, and invasion of LSCC. The subsequent results showed that IL-17 was highly expressed in neutrophil conditioned medium. Block of IL-17 could effectively inhibit the progression of LSCC induced by neutrophils. What is more, the results showed that IL-17 activated the JAK/STAT3 pathway in LSCC. Inhibition of the JAK/STAT3 pathway could significantly block neutrophil-induced LSCC progression. Our research reveals the complex interaction between neutrophils and LSCC cells, providing new ideas for the treatment of LSCC.

## 1. Introduction

Laryngeal squamous cell carcinoma (LSCC) is generated from the laryngeal mucosal epithelium. LSCC accounts for 2.4% of systemic malignancies and more than 90% of laryngeal carcinoma [1]. More than 90,000 people died of LSCC every year. LSCC patients suffer from severe impairments in breathing and swallowing, which cause great pain. More than half of the patients are diagnosed in an advanced stage [2]. LSCC patients' survival is seriously affected by local invasion and metastasis [3]. Therefore, it is of great importance to figure out the mechanism of LSCC progression and find new targets for therapy.

One of the important causes of LSCC patients' poor survival is metastasis [4]. However, the precise mechanisms leading to the metastasis remain unclear [5, 6]. Increasing evidences show that the microenvironment plays important roles in tumor progression [7, 8]. Immune cells, fibroblasts, and mesenchymal stem cells are important components of microenvironments [9]. Until recently, immune cells, especially macrophages, highlight the cancer research. However, with the development of new technologies, more and more functions of neutrophils in cancer are reported.

Neutrophils are the most prevalent type of immune cells, which are first responder cells to various inflammations and infections [10]. Neutrophils are reported to play important functions through every step of cancer progression, including initiation, growth, and metastasis [11]. Neutrophils could play diverse and even opposite functions in tumor progression [11–13]. Increasing evidence suggests that tumor-associated neutrophils could both promote and inhibit cancer progression [14, 15]. The neutrophil-to-lymphocyte ratio has been confirmed to be a predictor for LSCC poor survival [16, 17]. And tumor-infiltrating neutrophils are also correlated with poor LSCC prognosis [18]. But the specific mechanism of how neutrophils regulate prognosis of LSCC remains unclear. In this research, we want to investigate the crosstalk between neutrophils and LSCC.

## 2. Materials and Methods

### 2.1. Cell Culture

Tu177, Tu686, and HL-60 were maintained as described by previous reports [17, 19, 20]. Tu177 was obtained from Qincheng Biological Co., Ltd. Tu686 was from Tongpai Biological Technology Co., Ltd. HL-60 cells were obtained from ATCC (the Global Bioresource Center). Cells were cultured with RPMI-1640 medium containing 100 IU/mL of penicillin, 100 *μ*g/mL of streptomycin, and 10% of FBS. All the cells were cultured with 5% CO_2_ at 37°C.

### 2.2. Conditioned Medium from Neutrophils

HL-60 cells were seeded into cell culture dishes at the density of 4 × 10^3^ cells/mL. HL-60 cells were treated with 1.25% dimethyl sulfoxide (DMSO) (Sigma-Aldrich) for five days to form neutrophils. The control group was cultured without DMSO for five days.

At the sixth day, the neutrophils were cultured with fresh 1640 medium with 10% FBS and cultured for 24 hours at 37°C with 5% CO_2_. The cells and supernatants were separated by centrifugation at 2000g/min for 15 min. We collected the supernatants as the conditioned medium from neutrophils.

### 2.3. Conditioned Medium from LSCC Cells

Tu177 and Tu686 were seeded onto cell culture dishes at the density of 1 × 10^4^ cells/mL. Cells were cultured with 1640 medium with 10% FBS (Gibco, Invitrogen, USA) for 24 hours. The cells and supernatants were separated by centrifugation at 2000g/min for 15 min. We collected the supernatants for the conditioned medium. The conditioned media were filtered and stored until use at -80°C.

### 2.4. Western Blot

Cells are lysed with NETN150 (0.5% NP-40, 20 mM Tris (pH 8.0), 150 mM NaCl, and 6 mM EDTA). 25 *μ*g of proteins was separated by a 10% SDS-PAGE gel. Following electrophoresis, proteins were transferred to NC membranes and blocked with 5% nonfat milk. Then, we incubated the membranes with the indicated primary antibody. Protein levels were detected by western blot as previously described [21].

### 2.5. qRT-PCR

A TRIzol kit (Invitrogen, NY) was applied for RNA extraction following the instruction of the manufacturer. A reverse transcription kit (Invitrogen) was used for RNA reverse transcription. A SYBR kit (Roche) was used for qRT-PCR. The sequences of genes are shown in Table 1.

### 2.6. Proliferation Assay

4000 cells were seeded into 96-well plates per well. The cells were cultured with complete medium (control) or conditioned medium from neutrophils. The conditioned medium from neutrophils was added with or without the IL-17 antibody at the concentration of 200 *μ*M. We used the CCK8 kit (Dojindo, Kumamoto, Japan) for proliferation assays. We detected the absorbance at 450 nm at day 0, day 2, day 4, and day 6 following instructions of the manufacturer.

### 2.7. ELISA

IL-17 concentration was detected by using an ELISA kit (R&D Systems, USA) under the direction of the manufacturer's instruction.

### 2.8. Migration for Neutrophils

1.2 × 10^5^ HL-60-induced neutrophils were plated onto the upper chambers. Neutrophils which migrated into the lower chambers were collected and counted by using the Bio-Rad TC10 automatic cell counter 8 hours later. Each data was performed in triplicate, and the experiments were independently repeated three times.

### 2.9. Migration and Invasion Assays for LSCC Cells

24-well chambers (Corning, CA, USA) were used. 2 × 10^4^ cells with medium without FBS were added into the upper chamber. The down chambers were filled with conditioned medium or complete medium (control). The conditioned medium from neutrophils was added with or without 1 *μ*M IL-17 antibody (Novartis Cosentyx). The chambers were cultured at 37°C for 24 hours. The staining of the cells was performed as previously reported [15]. The chambers were pretreated with Matrigel for the invasion assay [15].

### 2.10. Drugs and Antibodies

The STAT3 inhibitor SH-4-54 was obtained from Selleck Company. Antibodies for vimentin, Snail, E-cadherin, p-JAK, JAK, p-STAT3, STAT3, and *β*-actin were all obtained from Cell Signaling Technology (Louis Park, MN, USA). The IL-17 antibody was from Novartis Cosentyx. Recombinant human IL-17 was from Sigma-Aldrich.

### 2.11. Statistical Analyses

All analyses were performed by using GraphPad Prism 8.0. Data were shown as mean ± S.D. The difference was determined by Student's *t*-test and analysis of variance. *P* < 0.05 was identified as statistically significant.

## 3. Results

### 3.1. LSCC Cells Enhance Neutrophil Activation and Migration

**Figure 1:**
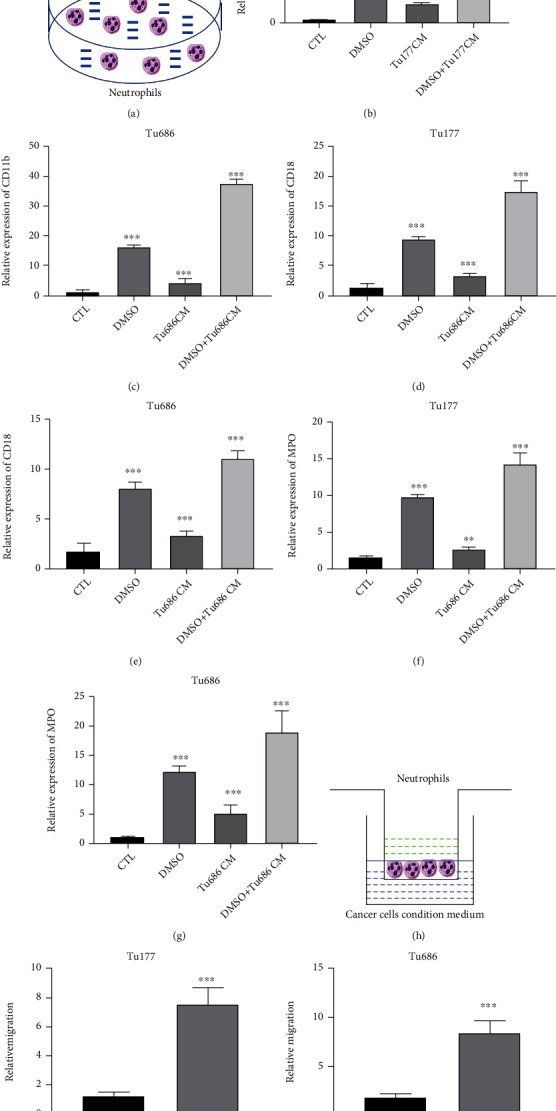
LSCC cells enhanced the activation and mobility of neutrophils. (a) Neutrophils were cultured with four different mediums: RPMI-1640 with 10% FBS (CTL), RPMI-1640 with 10% FBS and 1.25% DMSO (DMSO), conditioned medium from LSCC cells, or conditioned medium from LSCC cells with 1.25% DMSO. Six hours later, the neutrophils were collected. (b, c) qRT-PCR was used for CD11b level in neutrophils after treatment with Tu177- or Tu686-conditioned medium. (d, e) qRT-PCR was used for CD18 level in neutrophils after treatment with Tu177- or Tu686-conditioned medium. (f, g) qRT-PCR was used for MPO level in neutrophils after treatment with Tu177- or Tu686-conditioned medium. (h–j) Migration assays were carried out in neutrophils which were cocultured with Tu177- (i) or Tu686- (j) conditioned medium. The control medium was RPMI-1640 with 10% FBS. Data are shown of three independent experiments in triplicate (mean ± S.D.) (*n* = 3).

Neutrophils, as one of the most abundant immune cells, have been reported to play important roles in cancer progression. Firstly, we detected the influence of LSCC cells on neutrophils. The spatial and temporal regulations of *β*2 integrin CD11b/CD18 and Myeloperoxidase (MPO) are reported to play essential functions in activation and recruitment of neutrophils [22]. Signaling via adhesion molecules of the beta2 integrin family (CD11/CD18) plays important roles in polymorphonuclear leukocyte (PMN) recruitment and activation during inflammation [23]. MPO catalyzes the formation of reactive oxygen intermediates, including hypochlorous acid (HOCl). The MPO/HOCl plays an important role in microbial killing by neutrophils [24]. The neutrophils were cocultured with RPMI-1640 medium with 10% FBS, RPMI-1640 with 1.25% DMSO and 10% FBS, conditioned medium of Tu177 and Tu686, and conditioned medium of Tu177 and Tu686 with 1.25% DMSO, respectively. qRT-PCR analysis revealed that the conditioned medium of Tu177 and Tu686 could significantly upregulate CD11b, CD18, and MPO levels compared with the control group (Figures 1(a)–1(g)). These results suggested that LSCC conditioned medium could enhance the neutrophil activation. We also detected the influence of LSCC conditioned medium on neutrophil mobility. Transwell assays were used to detect the neutrophil mobility. 1640 with 10% FBS (control medium) or LSCC conditioned medium was added to the lower chamber. Transwell assays also indicated that the conditioned medium of Tu177 and Tu686 could promote the migration of neutrophils than the control medium (Figures 1(h)–1(j)). Together, LSCC cells could promote the activation and migration of neutrophils without direct interaction.

### 3.2. Neutrophils Promote Proliferation, Migration, and Invasion of LSCC

**Figure 2:**
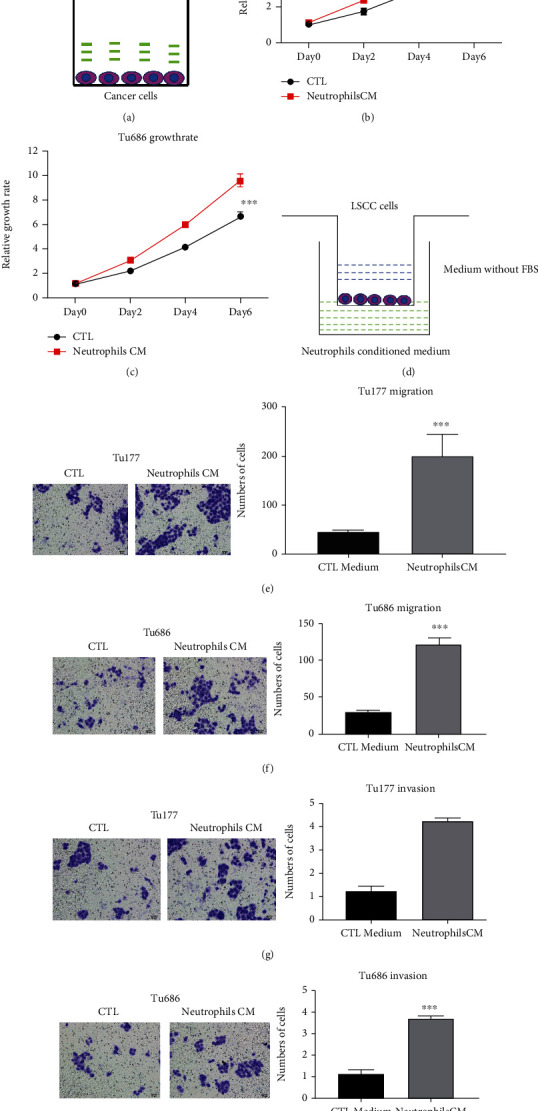
Neutrophils promoted proliferation, migration, and invasion of LSCC. (a) LSCC cells were cultured with neutrophil conditioned medium for the indicated days. The cells were cultured with RPMI-1640 with 10% FBS (control) or neutrophil conditioned medium. Growth rates were measured by CCK8. (b) Tu177 growth rates with different media. (c) Tu686 growth rates with different media. (d) Migration and invasion assays were performed as the figures showed. LSCC cells were added to the upper chamber. Conditioned medium was added to the lower chamber. The chambers were cultured at 37°C for 24 hours. (e, f) Migration results of Tu177 and Tu686 cells. (g, h) Invasion results of Tu177 and Tu686 cells.

Functions of neutrophils on LSCC were explored by the proliferation assay, migration assay, and invasion assay. LSCC cells were cocultured with neutrophil conditioned medium, and proliferation rates were detected by CCK8. The results showed that neutrophil conditioned medium promoted the proliferation rates of both Tu177 and Tu686 cells (Figures 2(a)–2(c)). LSCC cells were cocultured with neutrophil conditioned medium, and cell mobility was detected by the migration and invasion assay. Further experiments indicated that neutrophils also enhanced LSCC migration and invasion ability (Figures 2(d)–2(h)). Therefore, neutrophils play vital function in promoting proliferation, migration, and invasion of LSCC.

### 3.3. IL-17 Is Responsible for LSCC Progression Induced by Neutrophils

**Figure 3:**
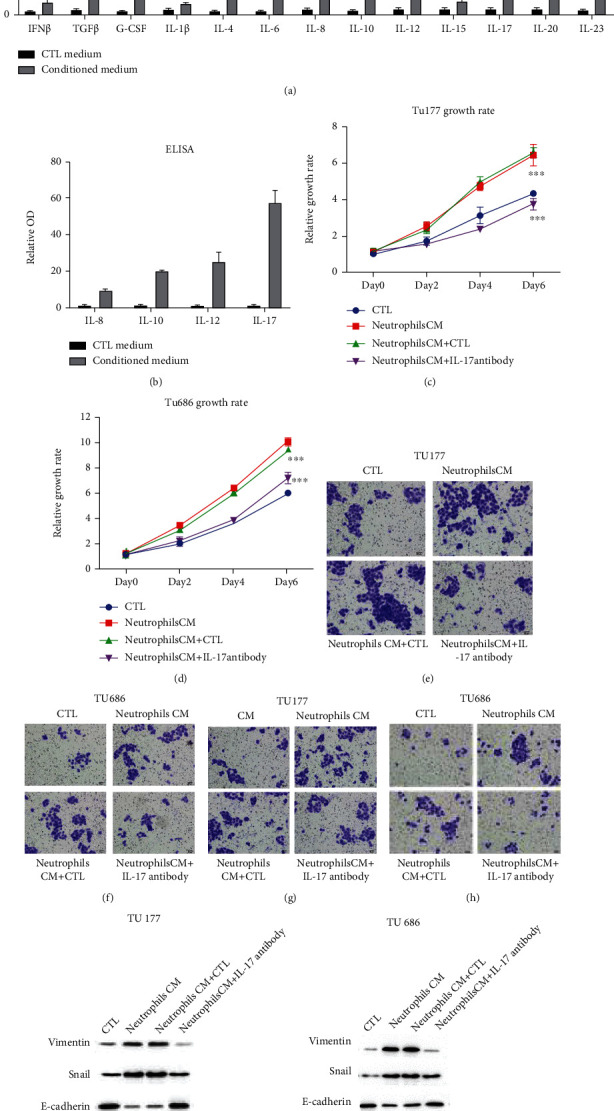
Neutrophils promoted LSCC progression through IL-17. (a) Neutrophil-associated inflammatory factor levels were detected in control medium and neutrophil conditioned medium by qRT-PCR. (b) ELISA was used for levels of IL-8, IL-10, IL-12, and IL-17 in control medium and neutrophil conditioned medium. (c, d) LSCC cells were cultured with control medium and neutrophil conditioned medium. The conditioned medium from neutrophils was added with or without 1 *μ*M IL-17 antibody (Cosentyx). Growth rates were detected by CCK8 in Tu177 and Tu686 cells. (e, f) The upper chambers were added with LSCC cells, and the lower chambers were added with conditioned medium with or without 1 *μ*M IL-17 antibody. Migration results of Tu177 and Tu686 were shown. (g, h) The upper chambers coated with Matrigel were added with LSCC cells, and the lower chambers were added with conditioned medium with or without 1 *μ*M IL-17 antibody. Invasion results of Tu177 and Tu686 were shown. (i, j) EMT-related markers in Tu177 and Tu686 cells were detected.

Increasing amounts of evidence show that neutrophils could promote cancer progression through inflammatory factors [25]. We detected the levels of inflammatory factors in neutrophil conditioned medium. Results showed that the conditioned medium contained INF*β*, TGF*β*, G-CSF, IL-1*β*, IL-4, IL-6, IL-8, IL-10, IL-12, IL-15, IL-17, IL-20, and IL-23 (Figure 3(a)). ELISA also confirmed the expression of IL-8, IL-10, IL-12, and IL-17 (Figure 3(b)). All results suggested that IL-17 was highly expressed in neutrophil conditioned medium.

To confirm whether IL-17 is the main factor contributing to the LSCC progression, we used the IL-17 antibody (Novartis Cosentyx) to block the function of IL-17. CCK8 assays showed that blockage of IL-17 could effectively weaken the proliferation rates of Tu177 and Tu686, which were enhanced by neutrophil conditioned medium (Figures 3(c) and 3(d)). Further studies also showed that IL-17 blockage could recede the migration and invasion ability induced by neutrophils (Figures 3(e)–3(h)). What is more, western blot results showed that neutrophils could promote the EMT (Epithelial-Mesenchymal Transition) of Tu177 and Tu686, and blockage of IL-17 could effectively inhibit the EMT (Figures 3(i) and 3(j)). All these experiments confirmed that IL-17 was responsible for neutrophil-induced LSCC progression.

### 3.4. Neutrophils Activate the JAK/STAT3 Pathway in LSCC Cells

**Figure 4:**
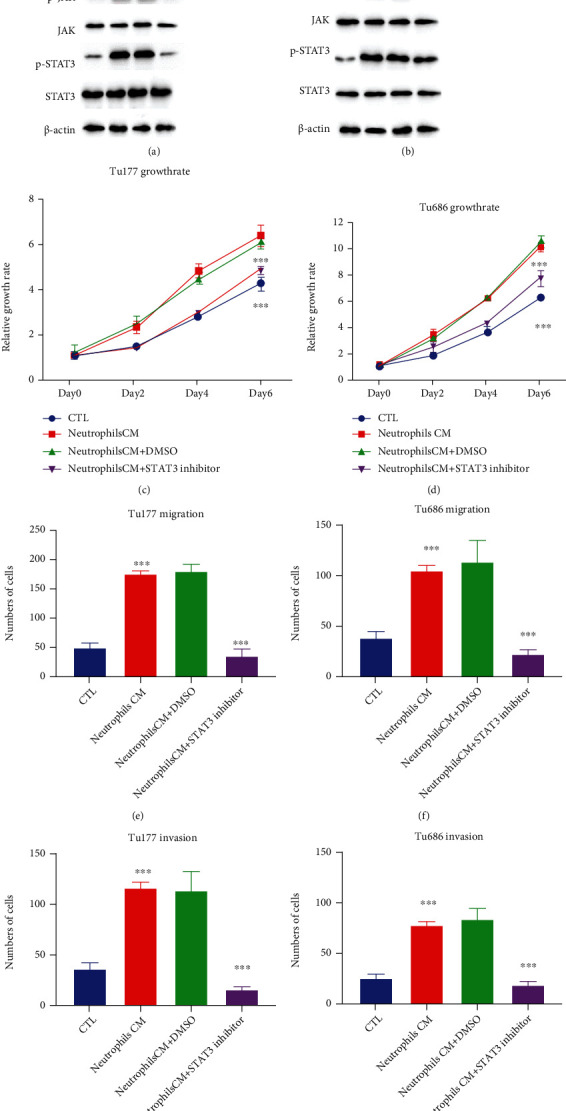
Neutrophils promoted LSCC progression through JAK/STAT3 activation. (a, b) Tu177 and Tu686 cells were cultured with the indicated medium. JAK and STAT3 activations were detected by western blot in Tu177 and Tu686 cells. (c, d) LSCC cells were treated with conditioned medium with or without the STAT3 inhibitor SH-4-54 (200 nM). We added DMSO equal to the volume of the SH-4-54 (dissolved in DMSO) in CM which we labeled as neutrophil CM+DMSO. Growth rates were detected by CCK8 in Tu177 or Tu686 cells. (e, f) LSCC cells were treated with conditioned medium with or without the STAT3 inhibitor SH-4-54 (200 nM). Migration results of Tu177 and Tu686 at 24 hours were shown. (g, h) LSCC cells were treated with conditioned medium with or without the STAT3 inhibitor SH-4-54 (200 nM). Invasion results of Tu177 and Tu686 at 24 hours were shown. Data are shown of three independent experiments in triplicate (mean ± S.D.) (*n* = 3).

JAK/STAT3 (the Janus kinase/signal transducer and activator of transcription 3) pathway activation has been found in various cancers. STAT3 (signal transducer and activator of transcription 3) is also reported to play important roles in LSCC chemoresistance, growth, and mobility [26–28]. We detected the activation of the JAK/STAT3 pathway by western blot. Our results showed that neutrophils activated the JAK/STAT3 pathway, and blockage of IL-17 could effectively reduce the activation of the JAK/STAT3 pathway (Figures 4(a) and 4(b)). To figure out the function of JAK/STAT3 in LSCC progression, we applied the JAK/STAT3 inhibitor in CCK8 (Figures 4(c) and 4(d)), migration (Figures 4(e) and 4(f)), and invasion assays (Figures 4(g) and 4(h)). Results exhibited that the STAT3 inhibitor effectively blocked neutrophil-induced LSCC proliferation, migration, and invasion (Figures 4(c)–4(h)).

### 3.5. IL-17/JAK/STAT3 Pathway Contributes to LSCC Progression

**Figure 5:**
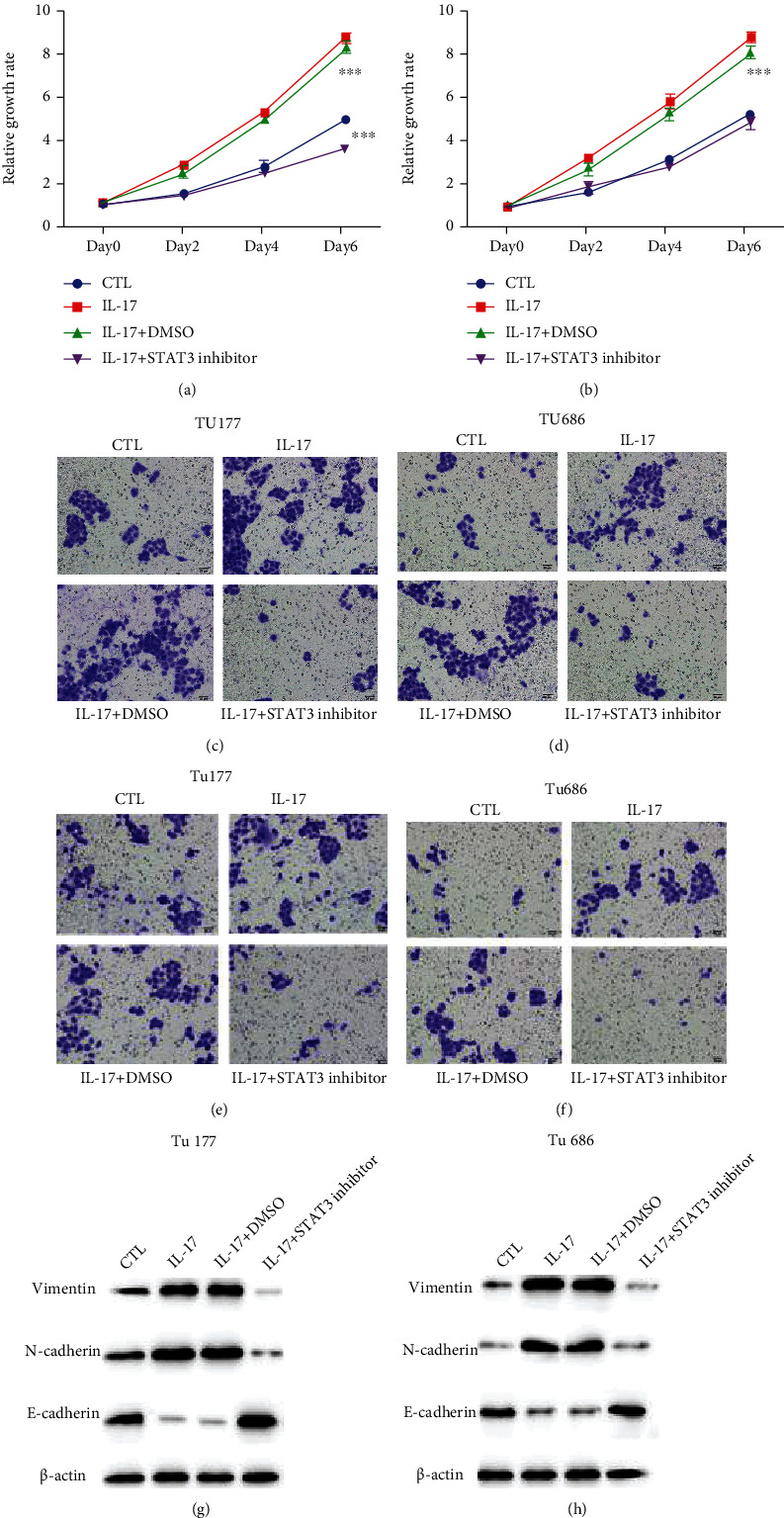
Inhibition of the IL-17/JAK/STAT3 pathway effectively blocked LSCC progression. (a, b) 2 ng/mL of IL-17 was added into the medium, and then, the cells were treated with SH-4-54 (200 nM). CCK8 assays were performed in Tu177 and Tu686 cells at day 0, day 2, day 4, and day 6. (c, d) Migration results of Tu177 and Tu686 at 24 hours were shown. (e, f) Invasion results of Tu177 and Tu686 at 24 hours were shown. (g, h) Tu177 and Tu686 cells were treated with 2 ng/mL of IL-17 or 200 nM STAT3 inhibitor SH-4-54 for 24 hours. EMT-related markers in Tu177 and Tu686 cells were detected by western blot.

We used IL-17 instead of neutrophil conditioned medium to confirm the function of the IL-17/JAK/STAT3 pathway in LSCC progression. CCK8 results showed that IL-17 played a similar role as neutrophil conditioned medium in promoting the proliferation of Tu177 and Tu686 (Figures 5(a) and 5(b)), and inhibition of STAT3 could effectively block the proliferation enhancement induced by of IL-17.

Further study showed that IL-17 also significantly enhanced LSCC migration and invasion, and JAK/STAT3 pathway inhibition effectively reduced the enhancement of mobility (Figures 5(c)–5(f)). What is more, western blot results showed that IL-17 also promoted EMT progression. E-cadherin was significantly downregulated with the addition of IL-17 and upregulated with the addition of the STAT3 inhibitor. Vimentin and Snail were found to increase with the addition of IL-17 and decreased when STAT3 activation was inhibited (Figures 5(g) and 5(h)). EMT-related marker changes might contribute to the enhanced migration and invasion abilities triggered by neutrophils. In summary, the experiments confirmed that the IL-17\JAK\STAT3 pathway was responsible for neutrophil-induced LSCC progression.

## 4. Discussion

LSCC is characterized by metastasis and recurrence, which lead to the poor survival of LSCC patients. The incidence of LSCC has gradually increased, while the development of LSCC treatment has stagnated [5]. LSCC has been a heavy burden for global health. Studying the specific mechanism of LSCC progression is of great value for LSCC diagnosis and treatment.

Immune cells are reported to play important roles in disease progression, especially cancer [29, 30]. Neutrophils account for 50% to 80% of leukocytes, which are critical factors in cancer microenvironment. The ratio of neutrophil to lymphocyte is an independent predictor for LSCC overall survival and progression-free survival [31, 32]. What is more, tumor-infiltrating neutrophils are found to promote LSCC progression [33]. However, how neutrophils infiltrate into tumor tissue and how neutrophils contribute to the LSCC progression still remain unclear. In this study, we tried to figure out the complex interaction between neutrophils and LSCC cancer cells. Our research showed that LSCC cells could promote neutrophil activation and mobility. And, in turn, neutrophils promoted the progression of LSCC. We uncovered the crosstalk between LSCC and neutrophils, which would provide new thoughts on LSCC researches.

The functions of IL-17 in cancer are reported to be controversial [34]. IL-17 has both tumor-promoting and tumor-suppressing functions [35]. IL-17 exerts tumor-promoting effects through enhanced signal transduction, angiogenesis, and tissue remodeling. IL-17 could stimulate tumor proliferation and self-renewal and promote tumor infiltration and angiogenesis by activating downstream transcription factors (STAT, NF-*κ*B, and AP1), antiapoptotic proteins (mTOR, Akt, Bcl-2, Erk, and Bax), and kinases (MAPK and HER1) [36, 37]. IL-17 also promotes cancer progression by changing the microenvironment of immune cells by cytokines and chemokines [38]. Interesting, IL-17 could also exert tumor-suppressing properties and correlate with better survival in various cancers such as chronic lymphocytic leukemia and gastric cancer [39, 40].

In our study, we found that neutrophils regulate the progression of LSCC through IL-17 secretion. And IL-17 exerts promoting functions in LSCC proliferation, migration, and invasion. We provided some new thoughts on LSCC target therapy.

STAT3 is a cytoplasmic transcription factor which belongs to the STAT family (signal transducer and activator of transcription family). STAT3 is reported to participate in various biological processes such as proliferation, mobility, and stemness [41, 42]. Hyperactivation of STAT3 is widely confirmed in numerous cancers and related to poor prognosis [43]. What is more, hyperactivation of STAT3 is found to regulate the immune microenvironment of the tumor [44] [45]. The JAK/STAT3 pathway is a potential target for proliferation, metastasis, chemoresistance, and immunity. In our research, we uncovered that IL-17 derived from neutrophils could activate the JAK/STAT3 pathway. And activation of JAK/STAT3 could promote the progression of LSCC. Our researches on the JAK/STAT3 pathway exhibit oncogenic roles in LSCC and might provide some new thoughts on LSCC therapy.

## 5. Conclusion

In conclusion, our research uncovered that LSCC cancer cells could activate neutrophils and promote the mobility of neutrophils. In return, neutrophils promoted proliferation, migration, and invasion of LSCC. Further study showed that neutrophils activated JAK/STAT3 in LSCC cells through secreting IL-17. Our research showed the complex crosstalk between neutrophils and LSCC, which would provide more thoughts on LSCC target therapy.

## Figures and Tables

**Table 1:** 

	Forward primer	Reverse primer
TGF-*β*	GGCCAGATCCTGTCCAAGC	GTGGGTTTCCACCATTAGCAC
IL-1	ATGATGGCTTATTACAGTGGCAA	GTCGGAGATTCGTAGCTGGA
G-CSF	TCCTGAACCTGAGTAGAGACAC	TGCTGCTTGTAGTGGCTGG
IL-4	GCCAAGACCCCTTCGAGAAAT	CCGATCCTGTTATCTGCCTCC.
IL-6	ACTCACCTCTTCAGAACGAATTG	CCATCTTTGGAAGGTTCAGGTTG
IL-8	TTTTGCCAAGGAGTGCTAAAGA	AACCCTCTGCACCCAGTTTTC
IL-10	GACTTTAAGGGTTACCTGGGTTG	TCACATGCGCCTTGATGTCTG
IL-12	ACCCTGACCATCCAAGTCAAA	TTGGCCTCGCATCTTAGAAAG
IL-15	TTGGGAACCATAGATTTGTGCAG	GGGTGAACATCACTTTCCGTAT
IL-17	TCCCACGAAATCCAGGATGC	GGATGTTCAGGTTGACCATCAC
IL-20	ATGAAAGCCTCTAGTCTTGCCT	GCCCCGTATCTCAGAAAATCC
IL-23	CTCAGGGACAACAGTCAGTTC	ACAGGGCTATCAGGGAGCA
GAPDH	CTGGGCTACACTGAGCACC	AAGTGGTCGTTGAGGGCAATG

qRT-PCR results were calculated by the method of 2^−ΔΔCt^, and glyceral-dehyde-3-phosphate dehydrogenase (GAPDH) served as the internal reference. The indicated gene expression was normalized to GAPDH.

## Data Availability

The data that support the findings of this study are available from the corresponding author upon reasonable request.
